# Regulation of Peripheral Inflammation by Spinal p38 MAP Kinase in Rats

**DOI:** 10.1371/journal.pmed.0030338

**Published:** 2006-09-05

**Authors:** David L Boyle, Toni L Jones, Deepa Hammaker, Camille I Svensson, Sanna Rosengren, Salvatore Albani, Linda Sorkin, Gary S Firestein

**Affiliations:** 1 Division of Rheumatology, Allergy, and Immunology, University of California San Diego School of Medicine, La Jolla, California, United States of America; 2 Department of Anesthesiology, University of California San Diego School of Medicine, La Jolla, California, United States of America; Leiden University Medical Centre, Netherlands

## Abstract

**Background:**

Somatic afferent input to the spinal cord from a peripheral inflammatory site can modulate the peripheral response. However, the intracellular signaling mechanisms in the spinal cord that regulate this linkage have not been defined. Previous studies suggest spinal cord p38 mitogen-activated protein (MAP) kinase and cytokines participate in nociceptive behavior. We therefore determined whether these pathways also regulate peripheral inflammation in rat adjuvant arthritis, which is a model of rheumatoid arthritis.

**Methods and Findings:**

Selective blockade of spinal cord p38 MAP kinase by administering the p38 inhibitor SB203580 via intrathecal (IT) catheters in rats with adjuvant arthritis markedly suppressed paw swelling, inhibited synovial inflammation, and decreased radiographic evidence of joint destruction. The same dose of SB203580 delivered systemically had no effect, indicating that the effect was mediated by local concentrations in the neural compartment. Evaluation of articular gene expression by quantitative real-time PCR showed that spinal p38 inhibition markedly decreased synovial interleukin-1 and −6 and matrix metalloproteinase (MMP3) gene expression. Activation of p38 required tumor necrosis factor α (TNFα) in the nervous system because IT etanercept (a TNF inhibitor) given during adjuvant arthritis blocked spinal p38 phosphorylation and reduced clinical signs of adjuvant arthritis.

**Conclusions:**

These data suggest that peripheral inflammation is sensed by the central nervous system (CNS), which subsequently activates stress-induced kinases in the spinal cord via a TNFα-dependent mechanism. Intracellular p38 MAP kinase signaling processes this information and profoundly modulates somatic inflammatory responses. Characterization of this mechanism could have clinical and basic research implications by supporting development of new treatments for arthritis and clarifying how the CNS regulates peripheral immune responses.

## Abstract 2

Inhibition of p38 MAP kinase in the CNS reduces peripheral inflammation and joint destruction in arthritic rats.

## Editors' Summary

**Background.:**

Rheumatoid arthritis is a disease marked by chronic inflammation, leading to joint pain and destruction. Pain and inflammation in the joints as well as other locations in the body (i.e., the “periphery”) are constantly monitored by the central nervous system (i.e., the brain and spinal cord). Scientists have long suspected that the central nervous system (CNS) can regulate inflammation and immune responses, but little is known about how the CNS does this. One potential player is a protein called p38 that is involved in a number of cellular processes critical to the development of rheumatoid arthritis. Several substances that block the action of p38 are effective in animal models of arthritis and are currently being tested in clinical trials in patients with rheumatoid arthritis. Originally, p38 was considered as a drug target that should mainly be blocked in the joints. But recent work has shown that pain in the periphery can lead to activation of p38 in the spinal cord, and that blocking p38 in the spinal cord might reduce peripheral pain.

**Why Was This Study Done?:**

Based on the observation that p38 is activated in the CNS in response to peripheral pain, the researchers who did this study wondered whether it might be involved in the interaction between inflammation in the joints and the CNS.

**What Did the Researchers Do and Find?:**

They induced inflammation in the joints of rats and then looked for responses in the spinal cord. They found that p38 was indeed activated in the spinal cord of these rats. This activation depended on another protein, called TNFα, which is another major regulator of inflammation. The scientists then blocked either p38 or the TNFα with drugs directly delivered to the spinal cord of the arthritic rats, they could substantially reduce inflammation, arthritis, and destruction of the joints, compared with rats that had undergone the same treatment but received no active drug. Treatment of arthritic rats with the same amount of drugs given directly under the skin (this is called “systemic treatment”) did not have any effect on the joints.

**What Do These Findings Mean?:**

Blocking p38 and TNFα by giving drugs systemically is known to have beneficial effects in animal models and human patients with rheumatoid arthritis. However, the drugs tested in patients to date also have side effects. Given that much lower doses were needed to achieve beneficial effects in the rats when the drugs were administered directly into the spinal cord, it is possible that spinal cord administration might reduce the side effects (and possibly the costs) of the drugs without compromising the benefits to the patients. If future studies confirm that the action of these drugs on the CNS is essential to achieve a response even when administered as a systemic treatment, designing drugs that get into the CNS easier might improve the effectiveness and/or make it possible to use lower doses systemically.

**Additional Information.:**

Please access these Web sites via the online version of this summary at http://dx.doi.org/10.1371/journal.pmed.0030338.

- MedlinePlus entry on rheumatoid arthritis
- Rheumatoid arthritis pages from the US National Institute of Arthritis and Musculoskeletal and Skin Diseases
- Rheumatoid Arthritis fact sheet from the American College of Rheumatology Description
- Wikipedia entry on rheumatoid arthritis  (note: Wikipedia is a free online encyclopedia that anyone can edit)

## Introduction

Central nervous system (CNS) regulation of peripheral inflammation may be a component of many immunological diseases, including rheumatoid arthritis. However, the mechanisms that allow the CNS to modulate peripheral inflammatory responses are largely unknown. For instance, neural mechanisms were recently described in which activation of glutamate receptors in the spinal cord dorsal horn alters skin and joint inflammation [1–3]. The spinal mechanisms, particularly the intracellular signaling pathways that integrate somatic afferent input and modulate peripheral inflammatory responses, have not been elucidated.

Recent data demonstrate that mitogen-activated protein (MAP) kinases, p38 in particular, can be activated in the spinal cord by peripheral noxious stimuli [4]. We considered that p38 might participate in reflexes that modulate peripheral inflammation. To test this hypothesis, expression and function of spinal p38 were evaluated in a model of chronic inflammation. We then tested whether TNFα serves as a key intermediary by activating spinal p38 in the same model. These pathways could potentially define a novel paradigm in which peripheral tissue inflammation is sensed by the CNS, most likely through somatic afferent pathways, resulting in p38 MAP kinase activation in spinal cord and subsequent modulation of peripheral responses.

## Methods

### Intrathecal Catheterization of Rats

Isoflurane-anesthetized Lewis rats (200–250 g) were implanted with an intrathecal (IT) catheter modified from the method previously described [5]. After a 6 d recovery period, all animals except those that appeared to have sensory or motor abnormalities (fewer than 5% of the total number) were used for experiments. For IT administration, 10 μl of drug or saline followed by 10 μl of isotonic saline was injected through the catheter. All animals were handled in accordance with USDA guidelines, and all procedures have been carefully reviewed and approved by the institutional animal subjects committee.

### Adjuvant Arthritis and Drug Treatment

Lewis rats were immunized at the base of the tail with 0.1 ml of complete Freund's adjuvant (CFA) on day 0 several days after catheter implantation (*n* = 6/group). Drug (p38 inhibitor SB203580 or vehicle) treatment was generally started on day 8 and continued daily until day 20. For etanercept experiments, drug was administered every 3 d. Clinical signs of arthritis generally begin on day 10, and paw swelling was determined every second day by water displacement plethysmometry. On the day of sacrifice, a probe with a 24 g bending force was applied to the lateral ankle of each hind paw for 4 s. Animals were graded (0/1) on whether they vocalized. Radiographs were obtained of the hind paws to assess bone changes by a semiquantitative scoring system (demineralization (0 to 2+), ankle and mid-foot erosions (0 to 2+), calcaneal erosion (0 to 1+), and heterotopic bone formation (0 to 1+) (maximum possible score, 6). Paws were fixed in formalin and decalcified for 2–3 d. Sections from paraffin-embedded tissue were stained with hematoxylin and eosin. A synovial inflammation score was determined using a semiquantitative scale that measures synovial inflammation, cartilage integrity, bone erosions, marrow infiltration, proteoglycan loss (in safranin O-stained sections), and extra-articular inflammation. All subjective assessments were made by an investigator blinded to the drug treatment.

### Immunostaining of Spinal Cords

Animals were deeply anesthetized with pentobarbital and perfused first with room-temperature 0.9% heparinized saline containing phosphatase inhibitors then with 4% chilled paraformaldehyde in 0.1 M phosphate buffer. Spinal cords were removed, postfixed for 12 h, and cryoprotected in 30% sucrose. The fixed tissue was mounted in Tissue-Tek ornithine transcarbamylase embedding compound, snap frozen, and stored at −80 °C. Sections (10 μm) were thaw-mounted onto Superfrost Plus Slides and air-dried for 30 min. Serial sections and/or double staining of the same section were used to determine colocalization of phosphorylated p38 (P-p38) (1:500, Cell Signaling Technology, Beverly, Massachusetts, United States) with cell markers OX42 (1:100, Biosource International, Camarillo, Texas, United States) for microglia and NeuN (1:1,000, Chemicon, Temecula, California, United States) for neurons. Sections were incubated at 4 °C for 24 h and washed three times for 10 min each in PBS. An ABC system (Vector Laboratories, Burlingame, California, United States) with Cy3-, Cy2-, or FITC conjugated secondary antibody were used for detection.

### Western Blot Analysis

Animals were anesthetized with 4% isoflurane and sacrificed, their spinal cords were removed by hydroextrusion, and the lumbar enlargement homogenized in lysis buffer (50 mM Tris, pH 7.4; 150 mM NaCl; 1 mM EDTA, pH 8; 0.5% Triton X-100), protease inhibitors and phosphatase inhibitors (Sigma, St. Louis, Missouri, United States). Insoluble material was removed by centrifugation at 14,000 *g* for 15 minutes at 4 °C. The protein concentrations in the extracts were determined using the DC protein assay reagent (Bio-Rad, Hercules, California, United States). Lysates were fractionated on Tris-glycine-buffered 10% SDS-PAGE and transferred to nitrocellulose membrane (Perkin-Elmer Life Sciences, Boston Massachusetts, United States). The membranes were blocked with Tris-buffered saline and 0.1% Tween 20 (TBS-T) containing 5% nonfat milk for 1 h at room temperature, followed by incubation with primary antibody at 4 °C overnight. After washing with TBS-T, the membrane was incubated with horseradish peroxidase-conjugated secondary antibody for 1 h at room temperature. Immunoreactive protein was detected with chemiluminescence and autoradiography (Perkin-Elmer), and analyzed using National Institutes of Health (NIH) Image software (version 1.61, NIH, Bethesda, Maryland, United States).

### Q-PCR

Ankle joints were excised and trimmed of skin. The tissue was then pulverized and RNA extracted using RNA-STAT-60 and standard techniques. The RNA was reversed transcribed into cDNA using random hexamer primers. TaqMan PCR amplification reactions were carried out in reaction volumes of 25 μl in MicroAmp Optical Plates and MicroAmp Optical Caps using the TaqMan Universal Master Mix with a Perkin Elmer 5700 thermal cycler. Each sample was analyzed in triplicate using 50 ng of cDNA in the reaction. Thermal cycling was initiated with an incubation at 50 °C for 2 min, followed by 95 °C for 10 min to activate the AmpliTaq polymerase, then 40 cycles of 95 °C for 15 s and 60 °C for 1 min. GADPH was used as a loading control for each sample. The standard curve method was used for quantification as previously described [6].

### CSF Collection and TNFα Assay

Halothane-anesthetized rats were placed in the prone position and the spinous processes at L1/L2 were identified as a tactile landmark. A midline skin incision, approximately 3 cm in length, was made caudally from the landmark to expose the interspinous space at L4/L5. The L4/L5 interspinous ligament and L5 spinous process were carefully removed. While elevating the L4 spinous process with forceps to widen the L4/L5 interlaminar space, the tip of a pulled capillary tube was obliquely introduced into the IT space. The jugular veins were compressed to increase the IT pressure and 40–50 μl of clear CSF was collected by capillary action. The CSF was transferred to microcentrifuge tubes, immediately frozen on dry ice and stored at −70 °C until assayed for TNFα by ELISA according to the manufacturer's instructions (R & D Systems, Minneapolis, Minnesota, United States).

### T Cell Proliferation Assay

Catheterized rats were immunized with CFA on day 0. Beginning on day 8, rats were treated with IT vehicle or 8 μg of SB203580. The animals were then sacrificed on day 14. Draining lymph node cells were isolated, washed with DMEM containing 2% FCS, and cultured in triplicate in 200 μl, U-bottom microtiter wells at 4 × 10^5^ cells per well with or without antigen in DMEM supplemented with 10% fetal bovine serum, penicillin, and streptomycin. L-glutamine and 5 × 10^−5^ M β-mercaptoethanol. Lymph node cells were tested for proliferation in response to ovalbumin at 1 μg/ml, HSP65 at 1 μg/ml, HSP65 180–188 peptide at 10 μg/ml, or Con A (2 μg/ml). Cells were incubated for 96 h at 37 °C in a humidified atmosphere of 5% CO_2_ [7]. Cultures were pulsed for the final 16 h with [^3^H]thymidine (Amersham; 1 μCi/well, specific activity 1 Ci/mmol), and thymidine uptake was measured using a liquid scintillation beta counter. Results are expressed as the mean cpm of triplicate cultures of stimulated cells minus medium control.

### Statistical Analyses

Means and standard errors of the mean are presented for all values. Data were analyzed by ANOVA followed by Dunnett's post-hoc test, the Student's t-test, or the Chi square test as appropriate.

## Results

### Phosphorylation of p38 MAP Kinase in the Spinal Cord in Adjuvant Arthritis

Initial experiments were performed to determine whether spinal p38 is phosphorylated in rats during the course of adjuvant arthritis (Figure 1). Rats were immunized with CFA on day 0 and sacrificed at various time points. Spinal cords were harvested and Western blot analysis was performed on the lumbar enlargements. As shown in Figure 1A, P-p38 levels increased in individual rats between days 8 and 17 after immunization. For comparison, COX2 expression was detected on day 5 and was elevated throughout the course of the model. To determine the location of P-p38-expressing cells, immunized rats were perfused on day 10, and tissue sections from the L4/L5 spinal cords were immunostained with anti-P-p38 antibody. Whereas dorsal horns of control rats contained only a few scattered P-p38-positive cells, numerous cells stained positively for P-p38 in the CFA-immunized rats (Figure 1B). These cells were present throughout the dorsal horn parenchyma, with especially dense staining in superficial laminae (especially lamina II). To determine which cell types express P-p38 in arthritic animals, double staining was performed using anti-P-p38 and antibodies specific for activated microglial cells (OX-42) or neurons (NeuN). Double staining with OX-42 showed (in merged images; see Figure 1E for a representative field and Figure 1I for an enlargement) that the majority of P-p38 staining was located in microglial cells. Double staining studies with NeuN also showed a limited number of neurons expressing P-p38 that were confined to lamina II (see Figure 1F–1H and 1J).

**Figure 1:**
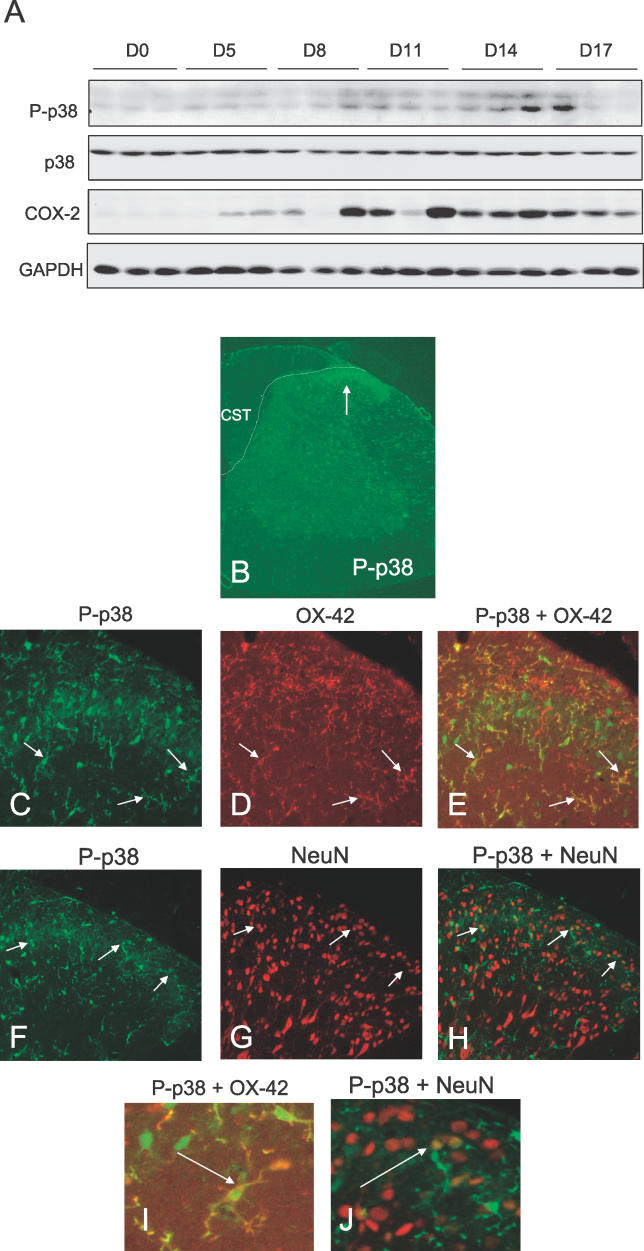
Phosphorylation of p38 MAP Kinase in the Spinal Cord (A) Western blot analysis of p38, P-p38, and COX2 in the spinal cord of rats with adjuvant arthritis. Rats were sacrificed from day (D) 0 through day 17. Note faint P-p38 staining on days 8 to day 17. GAPDH normalized expression = 0.22 ± 0.02 for prearthritis (days 0–5) and 0.32 ± 0.3 postarthritis (days 8–17; *p* = 0.0255). (B) Low-power view showing P-p38 expression in adjuvant immunized rats, especially in lamina II (CST, corticospinal tract). (C–E) High power view of P-p38 (C), OX-42 (D), and merged view (E) demonstrating expression of P-p38 in microglia of adjuvant immunized rats. (F–H) High-power view of P-p38 (F), NeuN (G), and merged view (H) demonstrating scattered neurons expressing P-p38 in adjuvant immunized rats. (I) and (J) show higher power view of (E) and (H), respectively. Arrow indicates positively staining cells in all photomicrographs.

### IT MAP Kinase Inhibitor Suppresses Adjuvant Arthritis

We then examined the effect of daily bolus injection of IT SB203580 (8 μg) on the clinical manifestations of adjuvant arthritis. As shown in Figure 2, paw swelling was markedly decreased in rats treated daily with IT SB203580 compared to either IT saline or the same dose of the inhibitor administered subcutaneously (SC) to catheterized rats. Rats with IT drug treatment did not respond to ankle pressure with vocalization (pain response), in contrast to rats with either IT saline or systemic drug administration (Table 1).

**Figure 2:**
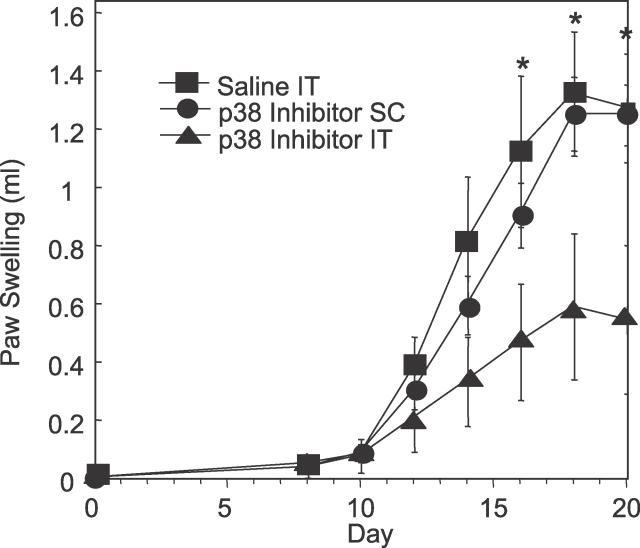
Effect of IT p38 Inhibitor on Paw Swelling in Adjuvant Arthritis Rats were immunized with CFA on day 0 and treated daily with IT p38 inhibitors (SB203580 at 8 μg/d), SC p38 inhibitor (SB203580 at 8 μg/d), or IT saline beginning on days 8–20. Paw swelling was measured by water displacement plethysmometry. The IT p38 inhibitor significantly decreased paw swelling (*p = 0.001 by ANOVA and Dunnett's post-ANOVA test; *n* = 5–6/group). This graph presents data from one of three separate experiments with similar results.

**Table 1:**
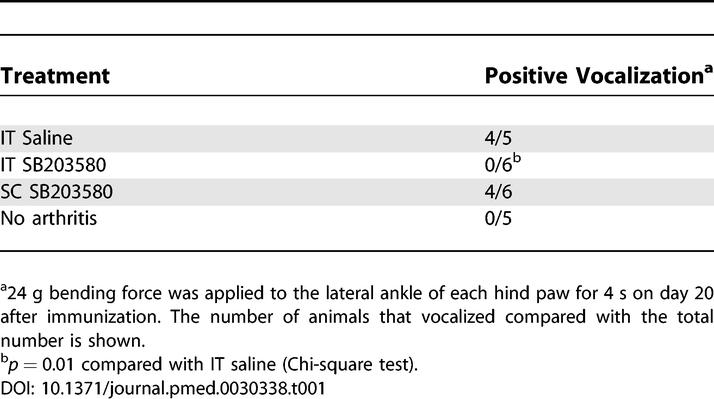
Behavioral Pain Effect of IT p38 Inhibitor in Adjuvant Arthritis

### IT MAP Kinase Inhibitor Suppresses Joint Destruction and Synovial Cytokine Gene Expression

Radiographic evidence of joint damage was also determined in the adjuvant arthritis model (Figure 3). IT treatment with the p38 inhibitor markedly decreased radiographic scores of joint damage, including bone erosions, cartilage loss, and demineralization, while systemic treatment with the same dose had no effect (Figure 3A). Representative radiographic images demonstrate the protective effect of IT SB 203580 (Figure 3B). The ankle histology in adjuvant arthritis also showed a trend toward less synovial inflammation, bone erosion, and cartilage loss in rats that received IT SB203580 (Figure 3C). The improvement in joint destruction after treatment with IT SB203580 suggests that the mediators and effector molecules in the joint might be affected by spinal p38 blockade. To evaluate this question, synovial gene expression of proinflammatory cytokines was determined using quantitative real-time PCR. Figure 3C shows that the IT p38 inhibitor significantly decreased synovial IL-1 (*p* = 0.025) and IL-6 (*p* = 0.006) mRNA in arthritis and there was a trend toward a decrease in TNFα mRNA (*p* = 0.089). Furthermore, expression of a key gene involved with extracellular matrix degradation (MMP3) was also decreased (*p* = 0.001; Figure 3D).

**Figure 3:**
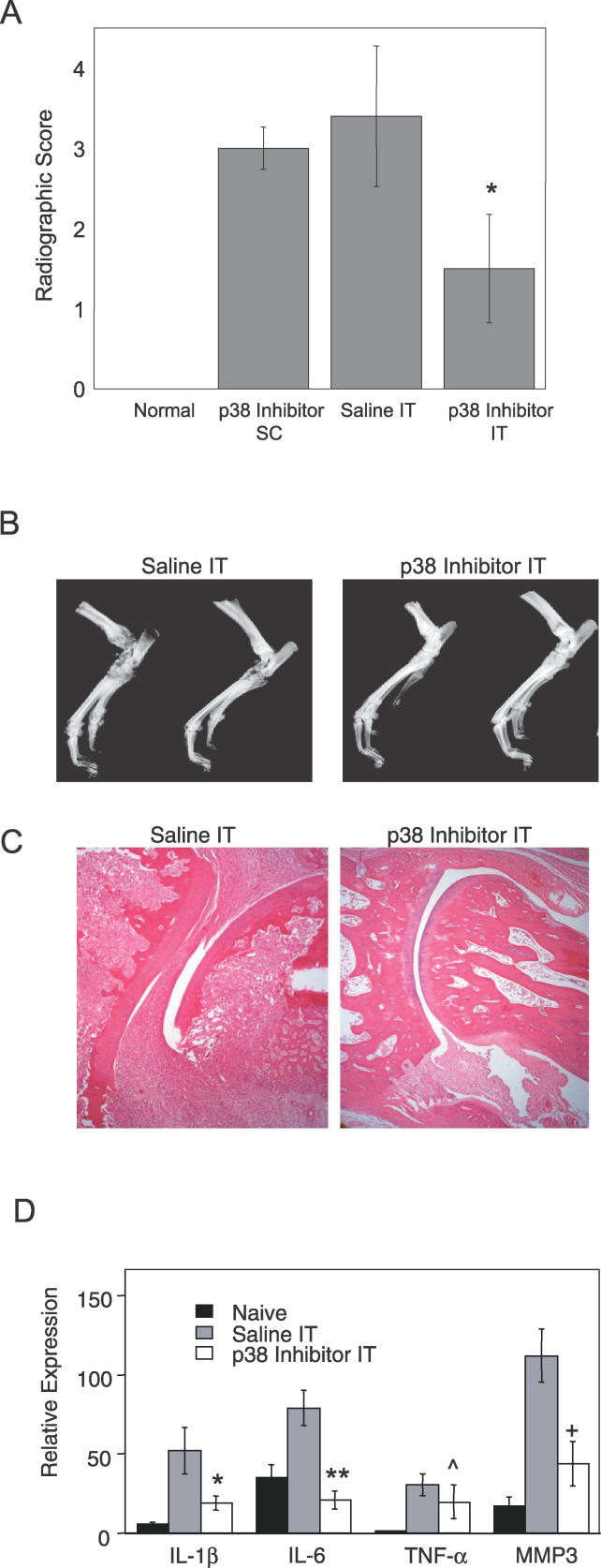
Effect of IT p38 Inhibitor on Joint Damage and Gene Expression in Adjuvant Arthritis Rats were immunized with CFA on day 0 and treated daily with IT SB203580 (8 μg) or IT saline beginning on days 8–20. (A) Radiographs of the hind paws were evaluated using a scoring system that demonstrated decreased damage in the SB203580-treated group compared with control rats. *p = 0.036 for IT SB203580 (*n* = 6/group). Similar results were observed in a separate experiment with treatment beginning on day 8 (*n* = 5–6/group). (B) Representative radiographs illustrate decreased joint destruction in the treated group. (C) Representative hematoxylin and eosin-stained sections demonstrate decreased erosions, cartilage damage, and synovial inflammation in the IT SB203580-treated group. (D) Quantitative real-time PCR was performed on the hind paws as described in Methods. Normal rat joints are also shown as a control. IT SB203580 significantly decreased IL-1 (*p = 0.025), IL-6 (**p = 0.006), and MMP3 (+*p* = 0.001) gene expression (*n* = 5–6/group); TNFα gene expression was modestly reduced, but did not reach statistical significance (^*p* = 0.089).

### IT SB203580 Does not Inhibit Peripheral T Cell Proliferation

In contrast to the effects on synovial cytokine expression, treatment of CFA-immunized rats with IT SB20580 had no effect on T cell proliferation. Table 2 shows the results of an experiment in which arthritic rats were treated from day 8 through day 14 with IT SB20580. Draining lymph node cells were harvested and cultured in the presence of epitopes implicated in adjuvant arthritis (HSP65 or the peptide HSP65 180–188, derived from HSP65) [8], ovalbumin, or the nonspecific mitogen Con A. The HSP peptides induced similar levels of proliferation in treated and control rats. No proliferation was observed using the control protein (ovalbumin), and no differences were observed between the groups stimulated with Con A.

**Table 2:**
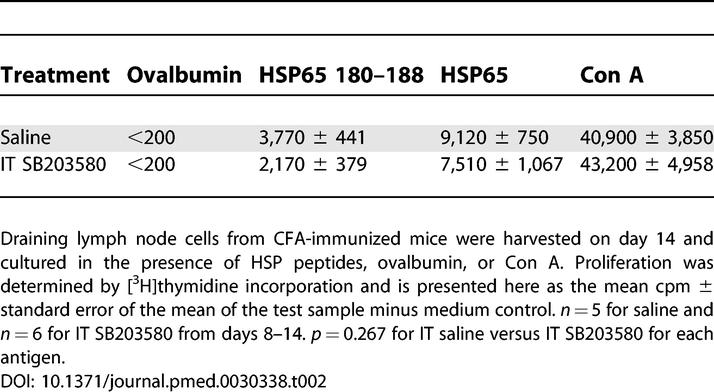
Effect of IT p38 Inhibitor on T Cell Proliferation in Adjuvant Arthritis

### Mechanism of p38 Activation: Role of TNFα

One possible mechanism of IT p38 blockade is through spinal TNFα, which is induced in the spinal cords of rats with adjuvant arthritis [9]. TNFα could potentially function upstream of p38 by activating kinases that phosphorylate p38, or it could be a downstream effect of p38 activation. If local TNFα is responsible for p38 activation and the subsequent proinflammatory effects, then spinal TNFα blockade in the CNS should theoretically suppress adjuvant arthritis and P-p38 levels. To test this hypothesis, we treated rats immunized with CFA with 100 μg or 300 μg of the TNF inhibitor etanercept IT every 3 d beginning on day 1. As shown in Figure 4A and 4B, etanercept significantly decreased paw swelling and joint destruction. Similar results were obtained in a second experiment when treatment was initiated on day 8 instead (unpublished data). Systemic treatment with the same doses of etanercept had minimal effect on paw swelling by day 20 (see Figure 4C). To determine if spinal TNFα is responsible for p38 activation, CFA-immunized rats administered either IT vehicle or etanercept on days 7 and 10 were sacrificed on day 11; immunostaining and Western blot analysis on these animals showed that etanercept decreased P-p38 expression, suggesting that TNFα acts upstream of p38 activation in this model (Figure 4D and 4E).

**Figure 4:**
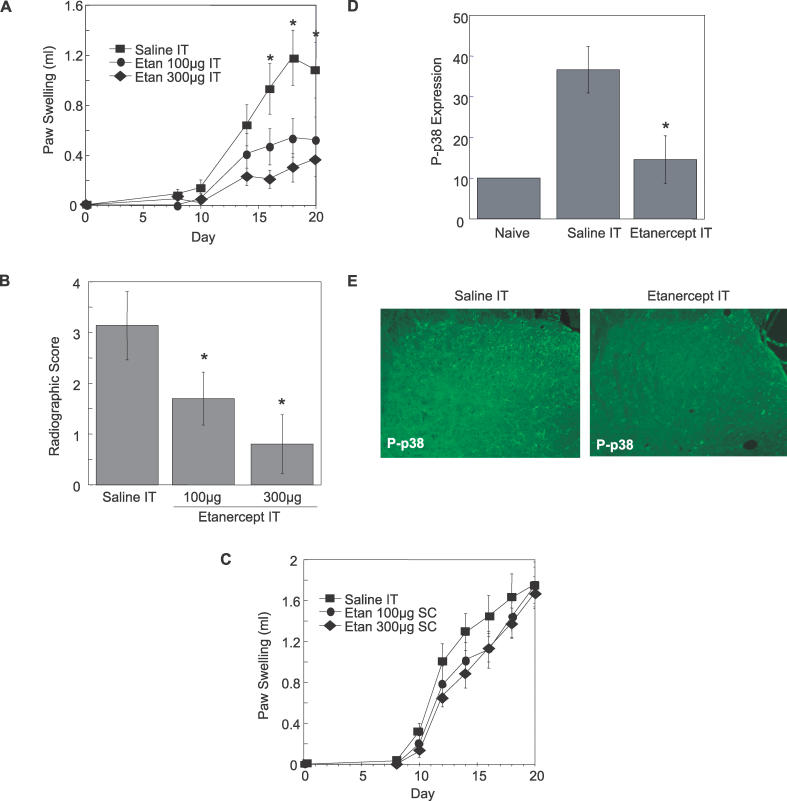
Effect of IT Etanercept on Adjuvant Arthritis Rats were immunized with CFA on day 0 and treated with IT etanercept (100 or 300 μg q.o.d.) beginning on day 1. (A) IT etanercept (Etan) significantly decreased paw swelling (*p = 0.001 for IT saline compared with either IT etanercept group) (*n* = 6/group). (B) IT etanercept significantly decreased joint damage (by radiographic score; *p = 0.026 for IT saline compared with either IT etanercept group) (*n* = 6/group). (C) Similar doses of etanercept given systemically (SC) beginning on day 1 had minimal effect on paw swelling (*p* = 0.398 for IT saline compared with either etanercept group) (*n* = 6/group). (D and E) IT etanercept decreased spinal P-p38 staining in adjuvant arthritis. Rats were immunized on day 0 and injected IT with 300 μg of etanercept or vehicle on days 7 and 10. They were sacrificed on day 11 and P-p38 was determined by Western blot (D) and immunofluorescence (E). Western blot data were normalized to GAPDH and demonstrated significantly lower levels of P-p38 after IT etanercept treatment (*n* = 6 for etanercept, 4 for saline, and 2 for naïve). *p = 0.023 compared with IT saline.

To determine if p38 also regulates TNFα expression in the spinal cord, a second series of experiments were performed to determine if IT SB203580 decreased TNFα levels in the CSF of catheterized rats with adjuvant arthritis. Rats were immunized on day 0 and treated with vehicle or IT SB203580 from days 8 to 14. CSF was collected and assayed for TNFα by ELISA. TNFα expression did decrease in response to the p38 inhibitor: In the vehicle-treated group, TNFα was 23.3 ± 11.0 pg/ml, whereas in the SB20358-treated group it was 7.6 ± 2.6 pg/ml (*p* = 0.038, *n* = 3 for saline and *n* = 4 for SB203580; TNFα < 0.5 pg/ml in normal rats).

## Discussion

Neural processing in the CNS can influence somatic host responses in the peripheral tissues, but the mechanisms remain poorly defined. The current study identifies an intracellular mechanism, to our knowledge not yet described, that involves spinal p38 MAP kinase as a link between the periphery and the CNS. Our results suggest that spinal blockade of p38 has profound effects on peripheral inflammation, arthritis, proinflammatory gene expression, and joint destruction.

The potential contribution of the spinal cord to peripheral inflammation has been explored in acute and chronic inflammation models, especially with agents that modulate glutamate and adenosine receptors [10]. Spinal administration of an adenosine A1 receptor agonist suppresses skin inflammation [2]. This action is independent of the sympathetic nerves and of dopaminergic activity, and it requires afferent input and/or dorsal root reflexes for the anti-inflammatory activity [11]. Other studies defining the influence of the CNS on peripheral inflammation have focused on vascular leakage rather than inflammatory cell recruitment. For instance, peripheral sympathectomy decreases paw edema in adjuvant arthritis, although it has no effect on neutrophil infiltration in carrageenan-injected skin [12,13]. Release of substance P at the site of inflammation probably accounts for primary effects on vascular integrity rather than neutrophil trafficking [14–16]. Studies in intra-articular carrageenan-induced inflammation models demonstrate that spinal non-*N*-methyl-D-aspartate (NMDA) glutamate receptor subtypes mediate the edema [1]. In contrast, neurogenic edema resulting from intradermal capsaicin injection is blocked by both non-NMDA and NMDA antagonists [17–19]. These contrasting results point to a high level of receptor activation specificity with variations in their peripheral effects depending on the type of inflamed tissue.

### MAP Kinase Activation in the Spinal Cord

While spinal glutamate receptor activation has been extensively evaluated in inflammation models, the role of intracellular signaling pathways such as those mediated by spinal MAP kinases is not known. MAP kinases in the peripheral tissues play a critical role in innate immunity and host defense by regulation of cytokines such as IL-1, IL-6, and TNFα and of enzymes involved in tissue remodeling, such as MMPs. Surprisingly, the same pathways are activated in spinal cord microglia and to a lesser extent in neurons when peripheral nerves are injured [20]. For instance, P-p38 increases in spinal cord within several hours of spinal nerve ligation (a standard model of neuropathic pain) [21]. MAP kinase blockade by spinal administration of a p38 inhibitor substantially reduces sciatic nerve ligation-induced mechanical allodynia. After unilateral chronic constriction injury to the mid-sciatic nerve or axotomy, spinal P-p38 is elevated for up to two weeks [22]. Spinal extracellular signal-regulated kinase (ERK) and c-Jun N-terminal kinase phosphorylation also peak in dorsal horn astrocytes three weeks after partial sciatic nerve ligation [23].

Data on activation of MAP kinases in the CNS due to inflammation are less well established. One study showed that carrageenan injection into rat foot pads induces a 5-fold increase in spinal ERK phosphorylation [24]. A similar increase in ERK activation occurs in an inflammatory pain model induced by injection of CFA into a rat paw [25]. Immunohistochemical studies show that both neurons and glia in the spinal cord contain the activated kinase. ERK activation after formalin injection appears to require metabotropic glutamate receptors rather than NMDA receptors [26]. Recent studies suggest that p38 is also activated in spinal cord by injection of carrageenan or formalin into the footpad [4,22], and inhibition of p38 reduces pain behavior associated with the peripheral inflammation. However, a study examining the spinal sequelae of a monoarthritis induced by intraplantar CFA reported no phosphorylation of p38 [27]. Another study reported that spinal administration of NMDA increased P-p38 in superficial dorsal horn, predominantly in microglial neurons, but also in a significant number of lamina II neurons [28]—much the same pattern that we observed in the current study with synovial inflammation.

### Spinal p38 Blockade Suppresses Arthritis

Based on the studies demonstrating that nerve injury is associated with MAP kinase activation in the spinal cord and that p38 blockade has antihyperalgesic activity in animal models, we explored the possibility of a corresponding effect on peripheral inflammation. We examined the activation state of p38 in the spinal cord during the evolution of chronic arthritis. Initial Western blot studies showed that p38 is phosphorylated in the spinal cord during the evolution of the adjuvant model. The kinase is activated in specific regions of the spinal cord dorsal horn within one week. Both neurons and microglial cells in the spinal cord contain P-p38, although double staining studies suggest that the latter is the primary location. A therapeutic intervention with IT SB203580, a commonly used p38 inhibitor, markedly decreased paw swelling and hyperalgesia. The amount required was a small fraction of the dose required if the same compound was administered systemically, and the same low dose given SC had no effect in arthritic rats. More striking, inhibition of p38 in the CNS also decreased synovial inflammation and radiographic evidence of bone and cartilage destruction. Expression of genes for proinflammatory cytokines and matrix metalloproteinases was also reduced by inhibition of p38 in the spinal cord.

Systemic p38 blockade has been extensively studied in inflammation and clearly demonstrates benefit in several models of arthritis [29–31]. The biological effects are similar to those observed in our studies evaluating IT administration of SB203580. Not only is synovial inflammation decreased, but joint damage is markedly attenuated by systemic p38 inhibition. One common observation is that relatively high doses of p38 inhibitors are often required when delivered by oral or parenteral routes, and the effective dose in vivo is often several hundred-fold higher than the amount required via IT therapy. These observations raise the intriguing possibility that the CNS might be responsible for a component of the anti-inflammatory effects seen with systemic administration of p38 inhibitors, and that inadequate CNS penetration contributes for this discrepancy.

### Spinal TNFα Blockade in Arthritis

The mechanism of central anti-inflammatory effects requires the expression of spinal or dorsal root ganglion TNFα. Noxious stimuli in the periphery, including adjuvant arthritis, enhance spinal TNFα release [9], and etanercept, a TNFα antagonist, inhibits allodynia and p38 activation in spinal cord when administered IT before spinal nerve ligation [32]. However, TNFα could either be the result of p38 phosphorylation or the cause of p38 activation in neurons and microglia in inflammation. Previous studies have documented that spinal TNFα expression is increased in the adjuvant arthritis model [9]. To evaluate the role of TNFα in the mechanism of IT SB203580 action, we administered etanercept IT in this model. Our data show that spinal TNF blockade mimics the anti-inflammatory and bone-protective effects of the p38 inhibitor. Furthermore, etanercept blocked spinal p38 phosphorylation, placing the cytokine upstream of the MAP kinase in the neural regulation of inflammation. The role of TNFα is probably more complex, because p38 can regulate production of this cytokine. Our data suggest that p38 inhibition also suppresses TNFα levels in the CSF of catheterized rats with adjuvant arthritis. The most likely explanation is that TNFα expression in the spinal cord both induces and is induced by p38 activation.

The mechanism by which the CNS modulates peripheral inflammation is not yet known. We have explored whether general or antigen-specific suppression of T cell function could account for our observations. T cell responses to epitopes implicated in the pathogenesis of adjuvant arthritis were not inhibited by treatment with an IT p38 inhibitor [8]. In other systems, the anti-inflammatory effect is independent of peripheral cytokines and is mediated by the release of adenosine in the peripheral tissue [2]. An efferent neural component rather than a circulating factor is responsible, because denervation inhibits the anti-inflammatory effects [11]. For instance, Tracey and colleagues have shown that parasympathetic outflow from the vagal nerve can suppress peripheral macrophage function and systemic inflammation via central muscarinic receptors [33]. Intraventricular treatment with CNI-1493, which inhibits p38, c-Jun N-terminal kinase, and perhaps other kinases, suppresses TNFα production and peripheral inflammatory responses [34]. The potential role of the parasympathetic nervous system in the adjuvant arthritis model is currently being investigated.

### Conclusions and Future Directions

Any study evaluating pain and inflammation in preclinical models has limitations, and this one is no exception. While adjuvant arthritis has many similarities to rheumatoid arthritis, no animal precisely replicates the human condition. There is also no guarantee that the pathways implicated in rodents have exact parallels to humans; this information can only be derived from clinical trials. Despite these issues, data implicating the CNS in peripheral inflammation could have relevance to the role of stress on immune responses, susceptibility to autoimmunity, and perhaps even placebo effects in patients.

We propose that inflammation in peripheral tissues is sensed by the CNS, most likely through somatic afferent pathways. This results in local TNFα production followed by p38 activation. The MAP kinase intracellular signaling pathway can subsequently relay information to the periphery, which is essential for full expression of somatic host responses. It is possible that this efferent flow occurs via activation of dorsal root reflexes in the somatic nerves, as has been shown for the vascular models; we are currently investigating the precise mechanism. Because glutamate receptor stimulation can increase spinal p38 phosphorylation [28], it is also possible that p38 links the previously described anti-inflammatory glutamate receptor mechanisms to the present study.

 In addition to providing insights on the interactions between the CNS and host defense, this novel MAP kinase-dependent pathway could influence the design of p38 inhibitors. Rather than focus exclusively on target organs for drug development, such as the synovium, or assiduously prevent entry into the CNS, it might be more appropriate to develop compounds that can penetrate the blood-brain barrier. This approach could offer the dual advantage of maximizing anti-inflammatory action and enhancing analgesic effects.

## Abbreviations

CFA: complete Freund's adjuvant
CNS: central nervous system
ERK: extracellular signal-regulated kinase
IT: intrathecal
MAP kinase: mitogen-activated protein kinase
NMDA: N-methyl-D-aspartate
P-p38: phosphorylated p38
SC: subcutaneous(ly)
TNF: tumor necrosis factor
